# Pramipexole Increases Go Timeouts but Not No-go Errors in Healthy Volunteers

**DOI:** 10.3389/fnhum.2016.00523

**Published:** 2016-10-18

**Authors:** Xue Qing Yang, Daniel Glizer, Andrew Vo, Ken N. Seergobin, Penny A. MacDonald

**Affiliations:** ^1^MacDonald Lab, Brain and Mind Institute, University of Western OntarioLondon, ON, Canada; ^2^Clinical Neurological Sciences, Schulich School of Medicine and Dentistry, University of Western OntarioLondon, ON, Canada

**Keywords:** pramipexole, motor impulsivity, Go No-go task, healthy subjects, basal ganglia, striatum

## Abstract

Parkinson’s disease (PD) is characterized by motor symptoms, such as resting tremor, bradykinesia and rigidity, but also features non-motor complications. PD patients taking dopaminergic therapy, such as levodopa but especially dopamine agonists (DAs), evidence an increase in impulse control disorders (ICDs), suggesting a link between dopaminergic therapy and impulsive pursuit of pleasurable activities. However, impulsivity is a multifaceted construct. Motor impulsivity refers to the inability to overcome automatic responses or cancel pre-potent responses. Previous research has suggested that PD patients, *on* dopaminergic medications, have *decreased* motor impulsivity. Whether effects on impulsivity are main effects of dopaminergic therapies or are specific to PD is unclear. Using a Go No-go task, we investigated the effect of a single dose of the DA pramipexole on motor impulsivity in healthy participants. The Go No-go task consisted of Go trials, for which keystroke responses were made as quickly as possible, and lesser frequency No-go trials, on which motor responses were to be inhibited. We hypothesized that pramipexole would *decrease* motor impulsivity. This would manifest as: (a) fewer No-go errors (i.e., fewer responses on trials in which a response ought to have been inhibited); and (b) more timed-out Go trials (i.e., more trials on which the deadline elapsed before a decision to make a keystroke occurred). Healthy volunteers were treated with either 0.5 mg of pramipexole or a standard placebo (randomly determined). During the 2-h wait period, they completed demographic, cognitive, physiological and affective measures. The pramipexole group had significantly more Go timeouts (*p* < 0.05) compared to the placebo group though they did not differ in percent of No-go errors. In contrast to its effect on pursuit of pleasurable activities, pramipexole did not increase motor impulsivity. In fact, in line with findings in PD and addiction, dopaminergic therapy might increase motor impulse *control*. In these patient groups, by *enhancing* function of the dorsal striatum (DS) of the basal ganglia in contrast to its effect on impulsive pursuit of pleasurable activities. These findings have implications for use and effects of pramipexole in PD as well as in other conditions (e.g., restless leg, dystonia, depression, addiction-related problems).

## Introduction

Parkinson’s disease (PD) is the second most common neurodegenerative disease worldwide (Hirtz et al., 2007). PD is age-related, with incidence increasing every year after age 55 (de Lau et al., 2004). The hallmark symptoms of PD are motor problems such as resting tremor, rigidity and bradykinesia (Jankovic, 2008). There are a number of non-motor symptoms of PD. Increasingly, researchers and clinicians are focusing on cognitive dysfunction in PD because these symptoms are most likely to lead to loss of independence and to functional disability (Aarsland et al., 2005). The mechanisms of cognitive dysfunction in PD are complex with some aspect even attributable to PD therapy (Cools et al., 2001; Rowe et al., 2008; MacDonald and Monchi, 2011; MacDonald et al., 2011).

A central pathological change in PD is degeneration of dopamine-producing neurons in the substantia nigra (SN), a brain structure located in the midbrain (Dauer and Przedborski, 2003). SN supplies dopamine principally to the dorsal striatum (DS; Dauer and Przedborski, 2003). The striatum is the input region of the basal ganglia, a collection of subcortical nuclei implicated in movement regulation as well as a number of cognitive functions. In fact, the motor symptoms of PD arise from DS dopamine depletion. DS has been implicated in functions such as motor planning, cognitive selections (Balleine et al., 2007), as well as in performing more considered and less habitual or pre-potent responses (Benke et al., 2003; Ali et al., 2009; Cameron et al., 2010; MacDonald et al., 2011, 2014; Mestres-Missé et al., 2012; Robertson et al., 2015). Another source of dopamine in the brain is the ventral tegmental area (VTA), adjacent to the SN (Haber and Fudge, 1997). VTA projections supply the ventral striatum (VS) of the basal ganglia, as well as temporal and prefrontal cortices, with dopamine (Haber and Fudge, 1997). VTA is relatively spared compared to SN and therefore motivational, cognitive and affective functions mediated by VTA-innervated brain regions are spared (Kish et al., 1988; Rakshi et al., 1999).

Dopaminergic therapies successfully treat PD motor symptoms. Levodopa and dopamine agonists (DAs; Dauer and Przedborski, 2003; Connolly and Lang, 2014) are the most effective therapies in PD. Levodopa is a dopamine precursor that is metabolized into dopamine in the brain (Lang and Lees, 2002). On the other hand, DAs such as pramipexole directly upregulate activity at post-synaptic dopamine receptors (Blandini and Armentero, 2014). Currently, both levodopa and DAs are titrated to address DS-mediated motor symptoms of PD (Connolly and Lang, 2014).

Despite the clearly positive effect on motor functions, the effect of DA therapies on cognitive symptoms in PD are complex (Cools, 2006; MacDonald and Monchi, 2011). Some cognitive functions are improved whereas others are impaired (Cools et al., 2001; Rowe et al., 2008; MacDonald and Monchi, 2011; MacDonald et al., 2011; Ganjavi and MacDonald, 2015). An important function that seems to be worsened by DA therapies is impulse control. In fact, this medication side effect can cause serious impulse control disorders (ICDs) that put patients at risk (Pontone et al., 2006; Weintraub et al., 2014). ICDs can manifest as pathological gambling, hypersexuality, overspending, hoarding and binge eating (Pontone et al., 2006; Weintraub et al., 2014). A study by Weintraub et al. (2010) found that as many as 13.6% of PD patients on DAs have an identified ICD. These disorders can have serious consequences. Although levodopa therapy can precipitate ICDs (Voon et al., 2010b; Weintraub et al., 2010), the percentage of patients experiencing this symptom on DAs is much higher (Driver-Dunckley et al., 2003; Pontone et al., 2006; Voon et al., 2006; Weintraub et al., 2006, 2010; Gallagher et al., 2007; Bostwick et al., 2009; Claassen et al., 2011; Garcia-Ruiz et al., 2014).

The vast majority of studies investigating effects of dopaminergic therapy on cognition have evaluated their effects in PD patients only (Thobois et al., 2010; MacDonald and Monchi, 2011; Poletti and Bonuccelli, 2013). It therefore remains unclear whether these results are main effects of dopaminergic therapy or whether these effects occur as an interaction between medication and PD pathophysiology. Studying the effects of dopaminergic therapy in healthy individuals can distinguish between these alternative explanations. The use of healthy young adults, in particular, provides an ideal control model for exploring the effects of dopaminergic medication on cognition. This strategy is advantageous because it avoids the significant variability in typical PD patient groups related to wide age ranges, as well as large differences in disease severity, medication doses and types. Studies with healthy controls also can rule out the possibilities that these medication effects on cognition occur only secondary to dopamine receptor sensitization through chronic exposure to dopaminergic therapy or to the fact that PD patients have reductions in dopamine transporter (DAT) levels. DAT clears and regulates dopamine at the synapse and reduced DAT levels could predispose to dopamine overdose effects (Harrington et al., 1996; Voon et al., 2009; Kordower et al., 2013; Kalia and Lang, 2015). Further, it is important to understand cognitive effects of dopaminergic therapy independent of PD because these medications are used in other conditions such as restless leg syndrome (Comella, 2002; Högl et al., 2006; Trenkwalder et al., 2008; Zintzaras et al., 2010; Hornyak et al., 2014) and in some cases of dystonia (Cloud and Jinnah, 2010; Jankovic, 2013). These treatments are also being explored for therapeutic effect in depression (Goto et al., 2006; Papakostas, 2006; Hori and Kunugi, 2012, 2013; Howland, 2012), drug addiction (Carroll et al., 1999; Streeter et al., 2005) and to address withdrawal symptoms (Ohmura et al., 2011; Makhinson and Gomez-Makhinson, 2014). Finally, if these cognitive effects are main results of dopaminergic therapy, this should alert clinicians to the possibility of cognitive improvements and impairments related to dopaminergic medications at any stage of PD as opposed to being more likely or marked with advancing disease or greater disease severity.

Impulsivity is not a unitary construct, however. Nombela et al. (2014) used factor analysis across different measures of impulsivity to identify four orthogonal impulsivity factors corresponding to measures of: (1) response conflict, interference and self-reported impulsivity; (2) motor inhibition; (3) time estimation and delay aversion; and (4) temporal discounting and reflection impulsivity. Additionally, Antonelli et al. (2011) have discussed impulsivity in terms of motivational/cognitive vs. performance/motor components. Cognitive impulsivity refers to behaviors such as riskier decision-making, impaired delay of gratification, increased pursuit of pleasurable activities and decreased feedback-based learning (Antonelli et al., 2011). This cognitive impulsivity seems to underlie the development of ICDs in PD. On the other hand, motor impulsivity is described as the inability to inhibit more automatic or pre-potent responses, as well as difficulty in canceling behaviors that have already been planned or initiated (Antonelli et al., 2011). PD patients with greater motor impulsivity are more susceptible to falls (Ahlskog, 2010; Wylie et al., 2012). In contrast to its effect of motivational/cognitive impulsivity, there is evidence that dopaminergic therapy might improve motor impulsivity in PD (Fera et al., 2007; Hiebert et al., 2014a; Caillava-Santos et al., 2015; van Wouwe et al., 2016). Our aim in the current study, was therefore to investigate the effect of DA on motor rather than cognitive impulsivity using the Go No-go paradigm (Simmonds et al., 2008; Wright et al., 2014).

Though the Go No-go paradigm is a prototypical assessment of motor impulsivity (Simmonds et al., 2008; Wright et al., 2014), only a small number of underpowered studies have investigated the effect of dopaminergic therapy on Go No-go performance in PD (Farid et al., 2009; Antonelli et al., 2014; Herz et al., 2014). We are aware of only one previous investigation of the effect of pramipexole in healthy volunteers on performance of the Go No-go task. Hamidovic et al. (2008) found that pramipexole treatment had no significant effect on Go No-go performance. However, participants were required to make decisions for eight number pairs, four of which were assigned as Go signals and the other four assigned as No-go signals. Multiple Go and No-go signals added complexity to the task and increased working memory load. Additionally, Hamidovic et al. (2008) presented Go and No-go signals at a 50:50 ratio, which would not generate pre-potency of the Go response.

In the present study, using the Go No-go task, we aimed to investigate the effect of dopaminergic therapy, the DA pramipexole in particular, on motor impulsivity (Rubia et al., 2001; Hamidovic et al., 2008; Antonelli et al., 2014) in a healthy, young control group, given the rationale and advantages discussed above. In contrast to the study conducted by Hamidovic et al. (2008), we sought to better isolate the effect of pramipexole on response withholding by employing one Go and one No-go signal, eliminating potential impacts of working memory or cognitive load. Further, we aimed to enhance motor impulsivity by adjusting the Go:No-go ratio to 75:25, to establish a strong pre-potent Go response. Understanding pramipexole’s effect on motor impulsivity will have implications for its use in PD as well as in other conditions such as restless leg, dystonia, depression and drug addiction.

## Materials and Methods

### Participants

Forty healthy young adults (16 males and 24 females, mean age 20.65 ± 1.12 years) were recruited at the University of Western Ontario. Participants were excluded if they had a history of neurological (e.g., stroke, seizures) or psychiatric conditions (e.g., clinical depression, hallucinations), a history of alcohol or drug abuse, or contraindications for pramipexole (e.g., monoamine oxidase inhibitors, iron supplements, cardiovascular disease). This study was approved by the Health Sciences Research Ethics Board (REB #102018) of the University of Western Ontario. All participants provided written informed consent before beginning the experiment in accordance with the Declaration of Helsinki (1991).

### Apparatus

The Go No-go task was performed on a 22.0″ monitor (LG Flatron W2242TQ) with a resolution of 1600 × 900 pixels and a desktop (LG model 73821B-10) using the Windows 7 Professional operating system. The screen was placed approximately 50 cm away from the participant. A keyboard (Logitech K120) was used to record participant responses.

### Procedures

**Figure 1:**
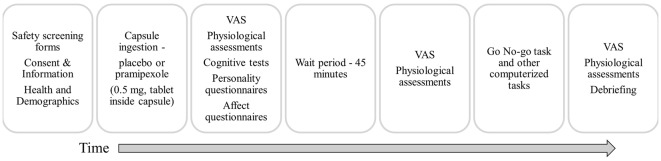
**Schematic outline of the experimental session.** VAS, Visual Analogue Scale (Bond and Lader, 1974).

Participants were instructed to abstain from caffeine, alcohol and nicotine on the testing day, to eat only light meals beforehand, and to abstain from food in the hour before the study to avoid interfering with pramipexole absorption. Participants were randomly assigned to receive either 0.5 mg of pramipexole or an equal volume of placebo in an identical capsule. The dose used here is consistent with previous studies conducted in healthy volunteers (Samuels et al., 2006, 2007; Drijgers et al., 2012). To ensure double-blindness, both placebo and pramipexole were administered orally in identical capsules that were prepared and assigned by a third-party not involved in data collection. Adverse effects were informally assessed by asking participants about their general well-being approximately every 15 min. Cognitive testing using the Go No-go task began 2 h after capsule administration to allow for optimal serum drug levels (Kirwin, 2007). At the conclusion of testing, participants were asked which capsule (placebo or pramipexole) they think that they received, and whether they were confident in their prediction. Participants were then debriefed about the details of the study and compensated for their time. A schematic outline of the procedure is shown in Figure 1.

#### Pre-Task Assessments

Participant demographic measures (age, sex, education, years of education, handedness) were obtained. Heart rate (HR) and both systolic and diastolic blood pressure (BP) were taken with an automated BP monitor (Omron model BP785N) at three different time points: Pre-Drug administration, Pre-Task (approximately 2 h post-drug administration) and Post-Task (approximately 3 h post-administration). Participants were given a self-reported visual analog scale (VAS) to assess subjective alertness (Bond and Lader, 1974) at each of the aforementioned time points as well. This was followed by a series of cognitive tests, including the (American National Adult Reading Test, ANART), Montreal Cognitive Assessment (MoCA), Controlled Oral Word Association Test (COWAT), as well as personality and affect questionnaires including the Barratt Impulsiveness Scale (BIS), Sensation Seeking Scale (SSS), Epworth Sleepiness Scale (Sleepiness), Oxford Happiness Scale (Happiness), Beck Depression Inventory II (BDI), Beck Anxiety Inventory (BAI) and Starkstein Apathy Scale (SAS). These personality and affect measures were administered only once during the experimental protocol since they are typically considered as trait measures and were unlikely to change due to pramipexole over the course of the experiment.

#### Go No-go Task

Commonly used to assess motor impulsivity, the Go No-go paradigm involves two trial types that are signaled by different visual stimuli. On Go trials, indicated by the target/Go visual stimulus, participants were required to respond with a keypress as quickly as possible. On No-go trials, signaled by the distractor/No-go visual stimulus, participants were instructed to withhold a keypress response (Rubia et al., 2001; Hamidovic et al., 2008; Antonelli et al., 2014). Our Go No-go task proceeded as follows: (i) a small fixation cross was presented in the center of the screen for 500 ms to signal the beginning of a trial; (ii) either a target/Go “X” or a distractor/No-go “K” stimulus appeared in the center of the screen for a maximum of 500 ms during which participants were required to make a keypress response for targets and withhold a keypress response for distractors; (iii) a blank screen was presented during the inter-trial interval (ITI) for 1000–1500 ms. The ITIs were modulated depending on the participant’s reaction time (RT) to maintain a constant length of 2000 ms between the beginning of subsequent trials. Participants were instructed to press the space bar on the keyboard for “X” Go stimuli and refrain from making any keystrokes for “K” No-go stimuli. The “X” was shown in 75% of trials, establishing “Go” as the pre-potent response. The “K” was shown in the remaining 25% of trials, randomly interspersed amongst the Go trials. Participants were asked to make their responses as quickly and accurately as possible. They were instructed that if their response was not made before the stimulus disappeared from the screen, this was deemed a timed-out, error trial. Trials were organized into four blocks of 64 trials each, with a 10 s break in between each block.

### Data Analysis

Four participants withdrew from the study due to nausea and dizziness. Their incomplete data was not included in the analysis. Demographic, cognitive and affective measures were analyzed between placebo and pramipexole groups using independent two-tailed *t*-tests. Physiological measures were compared between the two groups using mixed measures analysis of variance (ANOVA), with Medication status (Pramipexole vs. Placebo) as the between-subject factor and Time point (Pre-Drug vs. Pre-Task vs. Post-Task) as the within-subject variable. Percent errors in the Go trials and erroneous keypress responses in the No-go trials (No-go errors) were main dependent measures on a pair of independent sample *t*-tests. Whether participants failed to make a response (i.e., an omission error) or if they did not make a response within the deadline (i.e., timeout error), no response was recorded and therefore we could not distinguish between these types of errors. Go responses are quite straightforward. Further, Go trials accounted for 75% of trials, making the Go response pre-potent. We did not anticipate that young healthy participants would fail to respond entirely. Consequently, errors on Go trials are essentially synonymous with timeout errors. Lower numbers of timeouts on the Go trials and higher numbers of keypress responses in the No-go trials provide indicators of *more* impulsive responding. Percent of Go timeouts and of No-go errors were analyzed between the two groups with two-tailed independent *t*-tests. RTs were calculated in both the Go and No-go trials as the onset of the keypress response minus the onset of the visual stimulus (i.e., X or K) in milliseconds (ms). Correct No-go trials during which no keypresses were made were obviously excluded from the RT calculations. RTs of Go trials and No-go trials were analyzed between the two groups with two independent two-tailed *t*-tests. All data analysis was conducted using Excel (Version 2013), IBM SPSS Statistics (Version 21), and GraphPad Prism (Version 6). Significance values were set at *p* < 0.05.

## Results

### Demographic, Cognitive and Affective Measures

Measurements of various demographic, cognitive and affective variables were compared between participants treated with placebo or pramipexole (Table 1). Age, education, BDI, BAI, SAS, Happiness, Sleepiness, BIS, SSS, ANART, COWAT FAS Task, COWAT Animals Task and MoCA were analyzed with independent two-tailed *t*-tests. It is important to note that all of these measures were obtained *before* pramipexole effects, to establish baseline for both groups. No significant differences between the groups were found for any of the variables (*p* > 0.05 for all variables; age *t*_(38)_ = −0.84; education *t*_(38)_ = −0.49; BDI *t*_(38)_ = 0.78; BAI *t*_(38)_ = 0.50; SAS *t*_(38)_ = 0.23; Happiness *t*_(38)_ = −0.06; Sleepiness *t*_(38)_ = 0.42; BIS *t*_(38)_ = 1.00; SSS *t*_(38)_ = −0.15; ANART *t*_(38)_ = 0.14; COWAT FAS *t*_(38)_ = 0.46; COWAT Animal *t*_(38)_ = −0.14; MoCA *t*_(38)_ = 0.00). This establishes that there were no differences between our randomly assigned groups in cognitive ability, affective predisposition or other important demographic details such as age.

**Table 1:** **Demographic, cognitive and affective measures for healthy participants treated with either standard placebo or 0.5 mg pramipexole**.

	Treatment group	
Variable	Placebo	Pramipexole
*N*	20 subjects	20 subjects
Age	20.50 ± 1.28 years	20.80 ± 0.95 years
Sex	8 males, 12 females	8 males, 12 females
Education	15.40 ± 1.05 years	15.55 ± 0.89 years
Confidence	60% confident	45% confident
Prediction	75% correct	70% correct
BDI	9.60 ± 7.18	8.16 ± 5.38
BAI	8.60 ± 8.29	7.45 ± 6.18
SAS	11.50 ± 4.80	11.20 ± 3.44
Happiness	4.45 ± 0.58	4.47 ± 0.64
Sleepiness	10.05 ± 2.65	9.60 ± 3.94
BIS	62.05 ± 10.29	58.60 ± 11.41
SSS	19.80 ± 5.72	20.05 ± 4.47
ANART	118.95 ± 6.46	118.71 ± 4.44
COWAT FAS	40.15 ± 11.00 words	38.60 ± 10.61 words
COWAT animal	24.55 ± 1.12 words	24.80 ± 1.33 words
MoCA	27.8 ± 1.51	27.8 ± 1.94

*Values are presented as group means ± SEM. All values are in units of the respective questionnaire or task scale, unless otherwise stated (N = 20). Confidence: whether the subject could confidently predict which treatment they received; Prediction: whether the subject correctly or incorrectly predicted which treatment they received; BDI, Beck Depression Inventory II; BAI, Beck Anxiety Inventory; SAS, Starkstein Apathy Scale; Happiness, Oxford Happiness Questionnaire; Sleepiness, Epsworth Sleepiness Scale; BIS, Barratt Impulsiveness Scale; SSS, Sensation-Seeking Scale; ANART, American National Adult Reading Test; COWAT FAS, Controlled Oral Word Association Test FAS Task; COWAT Animal, COWAT Animal Task; MoCA, Montreal Cognitive Assessment. All factors were analyzed using two-tailed t-tests and no significant differences were found between the two groups (p > 0.05 for all factors)*.

### Physiological Measures

Physiological and alertness measures comprising HR, systolic BP, diastolic BP and VAS Alertness Scores were compared between placebo- and pramipexole-treated groups at the three time points with repeated-measures, two-way ANOVAs (Table 2).

**Table 2:** **Physiological measures for healthy participants treated with either standard placebo or 0.5 mg pramipexole**.

	Means ± SD
Variable	Placebo		Pramipexole
HR (beats per minute)		
Pre-Drug	76.158 ± 9.400		73.250 ± 12.298
Pre-Task	66.684 ± 9.434		68.750 ± 10.652
Post-Task	64.368 ± 8.902		67.850 ± 10.733
Systolic BP (mmHg)		
Pre-Drug	106.737 ± 8.723		112.650 ± 12.816
Pre-Task	102.421 ± 8.402		108.100 ± 12.473
Post-Task	101.947 ± 8.488		105.200 ± 25.816
Diastolic BP (mmHg)		
Pre-Drug	71.579 ± 9.459		72.850 ± 7.604
Pre-Task	65.368 ± 15.126		67.950 ± 7.075
Post-Task	67.789 ± 5.170		69.500 ± 7.931
VAS alertness score		
Pre-Drug	62.678 ± 12.219		64.422 ± 14.429
Pre-Task	58.639 ± 17.700		48.550 ± 15.475
Post-Task	56.761 ± 19.312		45.311 ± 14.931

**Variable**	***F* stat**	***P*-value**	**Comparisons**

HR (beats per minute)			
Medication effect	*F*_(1,37)_ = 0.08	*p* = 0.774	
Time effect	*F*_(2,74)_ = 32.40	*p* < 0.001	Pre-Drug > Pre-Task ***, Pre-Drug > Post-Task ***
Medication × Time Interaction	*F*_(2,74)_ = 4.37	*p* = 0.016	Placebo: Pre-Drug > Pre-Task ***, Pre-Drug > Post-Task ***
			Pramipexole: Pre-Drug > Pre-Task *, Pre-Drug > Post-Task **
Systolic BP (mmHg)			
Medication effect	*F*_(1,37)_ = 1.812	*p* = 0.185	
Time effect	*F*_(2,74)_ = 3.54	*p* = 0.034	Pre-Drug > Pre-Task **
Medication × Time interaction	*F*_(2,74)_ = 0.192	*p* = 0.825	
Diastolic BP (mmHg)			
Medication effect	*F*_(1,37)_ = 0.739	*p* = 0.395	
Time effect	*F*_(2,74)_ = 5.167	*p* = 0.008	Pre-Drug > Pre-Task *, Pre-Drug > Post-Task *
Medication × Time interaction	*F*_(2,74)_ = 0.073	*p* = 0.930	
VAS alertness score			
Medication effect	*F*_(1,37)_ = 2.628	*p* = 0.113	
Time effect	*F*_(2,74)_ = 13.650	*p* < 0.001	Pre-Drug > Pre-Task ***, Pre-Drug > Post-Task ***
Medication × Time interaction	*F*_(2,76)_ = 4.111	*p* = 0.020	Pramipexole: Pre-Drug > Pre-Task ***, Pre-Drug > Post-Task ***

Effect of treatment with either standard placebo or 0.5 mg pramipexole on HR (beats per minute), systolic blood pressure (BP; mmHg), diastolic BP (mmHg) and visual analog scale (VAS) drowsiness score (*N* = 20). Data represent group means ± SD. All factors were analyzed with repeated-measures two-way ANOVAs. Measurements were taken Pre-Drug, Pre-Task (2 h after treatment) and Post-Task (3 h after treatment). A significant main effect of time was found for heart rate (HR; ***p < 0.001), with Bonferroni multiple comparisons analysis showing decreased HR from Pre-Drug to Pre-Task (***) and from Pre-Drug to Post-Task (***). The main effect of time was qualified by the time-medication interaction effect, with both placebo and pramipexole groups showing significantly decreased HR from Pre-Drug to Pre-Task and Pre-Drug to Post-Task (all *p* < 0.05). No significant main effect of medication was found. A significant main effect of time was found for systolic BP (*p < 0.05), with Bonferroni multiple comparisons analysis showing decreased systolic HR from Pre-Drug to Pre-Task (**p < 0.01). No significant main effects of medication or time-medication interaction effects were found. A significant main effect of time was found for diastolic BP (**), with Bonferroni multiple comparisons analysis showing decreased diastolic BP from Pre-Drug to Pre-Task (*) and from Pre-Drug to Post-Task (*). No significant main medication effects or time-medication interaction effects were found. A significant main effect of time was found for VAS Alertness Score (***), with further Bonferroni multiple comparisons analysis showing decreased VAS Alertness Score from Pre-Drug to Pre-Task (***) and from Pre-Drug to Post-Task (***). The main effect of time was qualified by the time-medication interaction effect, with the pramipexole group showing significantly decreased VAS Alertness Score from Pre-Drug to Pre-Task (***) and from Pre-Drug to Post-Task (***). No significant main effect of medication was found.

#### Heart Rate

HR showed a significant main effect of Time (Table 2; *F*_(2,74)_ = 32.40, *p* < 0.001), with decreases in HR from Pre-Drug to Pre-Task (*p* < 0.001) and Pre-Drug to Post-Task (*p* < 0.001). There was also a Time × Medication interaction effect (*F*_(2,74)_ = 4.37, *p* = 0.016). Bonferroni-corrected pairwise comparisons revealed that for both placebo and pramipexole groups, HR decreased significantly from Pre-Drug to Pre-Task and from Pre-Drug to Post-Task (all *p* < 0.05), though this difference was greater in the placebo than in the pramipexole group.

#### Blood Pressure

Systolic BP showed a significant main effect of Time (*F*_(2,74)_ = 3.54, *p* = 0.034) with Bonferroni-corrected pairwise comparisons revealing a significant decrease in systolic BP from Pre-Drug to Pre-Task (*p* = 0.005). However, systolic BP showed no main Medication effects or Time × Medication interaction effects. Diastolic BP also showed a significant main effect of Time (*F*_(2,74)_ = 5.167, *p* = 0.008) with Bonferroni-corrected pairwise comparisons revealing significant decreases in diastolic BP from Pre-Drug to Pre-Task (*p* = 0.035) and from Pre-Drug to Post-Task (*p* = 0.034). No main Medication effects or Time × Medication interaction effects were found for diastolic BP.

In summary, physiological measures indicated that both HR and BP decreased over time. With regards to HR, the placebo group showed a larger decrease than the pramipexole group.

### Alertness

VAS Alertness was found to have a significant main effect of Time (*F*_(2,74)_ = 13.650, *p* < 0.001) with Bonferroni-corrected pairwise comparisons revealing significant decreases in VAS Alertness Score from Pre-Drug to Pre-Task (*p* = 0.001) and from Pre-Drug to Post-Task (*p* < 0.001). The main effect of Time was qualified by a Time × Medication interaction effect (*F*_(2,76)_ = 4.111, *p* = 0.02), with significantly greater decreases in the pramipexole group from Pre-Drug to Pre-Task (*p* < 0.001) and from Pre-Drug to Post-Task (*p* < 0.001). No significant main effect of Medication was found, however.

These decreases in physiological and alertness measures were not surprising, because participants were sitting, inactive, and becoming more comfortable and habituated to the experimental setting throughout the 3-h study period.

### Behavioral Go No-go Measures

**Figure 2:**
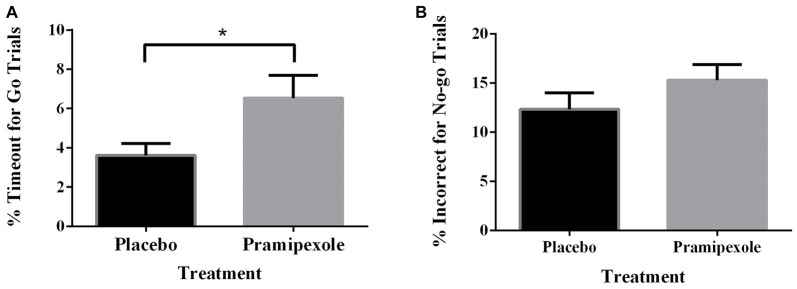
**Effect of treatment with either standard placebo or 0.5 mg of pramipexole on Go trial timeouts and No-go trial errors (*N* = 20).** Both variables were analyzed with two-tailed *t*-tests. Data represent mean ± SEM. **(A)** Participants treated with pramipexole timed out in a significantly greater number of Go trials compared to participants treated with placebo (*p < 0.05). **(B)** No significant difference was found for number of No-go trial errors between the placebo- and pramipexole-treated participants (*p* > 0.05).

**Figure 3:**
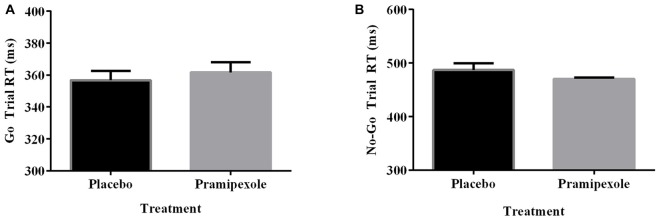
**Reaction time (RT; ms) for Go trials and No-go trials during the Go No-go task for healthy participants treated with either standard placebo or 0.5 mg pramipexole (*N* = 20).** Both variables were analyzed with two-tailed *t*-tests. Data represent mean ± SEM. **(A)** No significant differences between placebo and pramipexole groups were found for Go trial RT (*p* > 0.05). **(B)** No significant differences between placebo and pramipexole groups were found for No-go trial RT (*p* > 0.05).

Percent of Go timeouts and No-go errors were analyzed with two-tailed independent *t*-tests. Participants treated with pramipexole had a significantly higher percent of Go timeouts (*t*_(38)_ = −2.265, *p* = 0.029) compared to those treated with placebo (Figure 2A). Percent of commission errors on No-go trials was not significantly different between the placebo and pramipexole groups (Figure 2B, *t*_(38)_ = −0.675, *p* = 0.504). Overall RTs of Go trials and No-go trials were analyzed between placebo and pramipexole groups using two-tailed *t*-tests. Neither Go trial RT (Figure 3A, *t*_(38)_ = −0.574, *p* = 0.569) nor No-go trial RT (Figure 3B, *t*_(38)_ = 1.315, *p* = 0.196) showed significant differences between placebo and pramipexole groups, revealing that Go trial timeouts were not simply related to a general tendency toward slower responding in the pramipexole group.

## Discussion

### Summary of Findings

In this study, we found that the percentage of No-go errors was not increased for participants treated with pramipexole relative to those treated with placebo. However, the pramipexole group had significantly more timeouts in the Go condition compared to their placebo-treated counterparts. This pattern of findings suggests that pramipexole does not enhance motor impulsivity, in contrast to its detrimental effect on cognitive impulsivity and potential for producing ICDs in PD (Cools et al., 2003; Riba et al., 2008; Voon et al., 2010a; Antonelli et al., 2014). The finding of more timeout errors in the Go condition might suggest that pramipexole leads to *less* impulsive responding. Lower motor impulsivity or evidence of a more conservative criterion for responding was not replicated for pramipexole-treated participants in the No-go condition though. There were no between-group differences in terms of No-go errors. There were far fewer No-go than Go trials, however, and in this way, the Go timeout measure was potentially better powered to detect a subtle biasing effect of pramipexole on impulsivity.

The equivalent RTs between the placebo and pramipexole groups showed that the increased number of Go timeouts was not simply due to impaired motor function or generally slowed processing related to pramipexole. Further, although there were decreases in HR, systolic BP, diastolic BP and VAS Alertness Score across the experiment, there were no main effects of Medication on these measures. We attribute these changes in our physiological measures to increased comfort with the testing situation as well as to physical inactivity during the testing session. A significant Time × Medication interaction on VAS, suggests that the pramipexole group rated themselves as having greater differences in alertness from baseline to Pre- and Post-Task measures than the placebo group. However, entirely equivalent RTs between groups makes it less likely that our finding of increased Go trial timeouts was attributable to decreased alertness alone. Further, even at the very end of the experiment, the Pramipexole group’s average VAS score hovered around the neutral mark, indicating that they were feeling neither very sleepy nor very alert.

Finally, our results were not due to pre-existing group differences in impulsivity, demographic, cognitive or affective factors. Baseline measures of these factors were not significantly different between groups.

### Previous Studies of Dopaminergic Therapy on Go No-go Performance

Here, we replicate the finding of Hamidovic et al. (2008) that pramipexole had no effect on No-go errors in healthy controls. At odds with their results, however, we found significantly more timeout errors in the pramipexole relative to placebo groups, hinting at the possibility that pramipexole does not enhance but potentially reduces motor impulsivity. As previously discussed, Hamidovic et al. (2008) task differed from ours in a number of critical ways possibly accounting for slightly different findings. They employed multiple Go and No-Go signals, confounding the measure of motor impulsivity with working memory and cognitive load. Further, in their study, Go and No-go trials were presented in equal proportion. By using one Go and one No-go stimulus, we eliminated potential effects related to memory load, better isolating motor impulsivity. To enhance the difficulty of withholding a response, we presented Go and No-go trials in a ratio of 75:25, establishing a pre-potent Go response. To our knowledge, no other studies have investigated the effect of pramipexole on healthy controls in a standard Go No-go paradigm as we have performed here. Ours is also the first to find the suggestion of less impulsive responding in the Go condition in healthy participants.

Only a small number of studies have directly investigated the effect of dopaminergic therapy on Go No-go task performance in PD patients. Comparing performance *on* vs. *off* pramipexole in PD patients (*N* = 7), Antonelli et al. (2014) found that medication had no effect on motor impulsivity in a simple Go No-go task. Farid et al. (2009), also employed a simple Go No-go paradigm and tested patients *on* and *off* levodopa (*N* = 9) relative to healthy controls (*N* = 9). They found no differences in RT or overall task accuracy between healthy controls and patients, and there were no differences related to levodopa. It is important to note that PD patients were tested twice whereas the healthy controls were tested only once. The results confounded as patients might have experienced practice effects during the second testing session, which was always performed in the *on* state. Another study, by Herz et al. (2014), used a more complex Go No-go paradigm involving three stimuli. They found no RT or accuracy differences between PD patients with and without dyskinesia, whether they were tested *on* or *off* medication (*N* = 13, 13), relative to healthy controls (*N* = 13). Due to the added complexity of their paradigm, in which they were asked to press left or right keys or to withhold a response, the task might not have established the necessary pre-potent response.

In general, the small number of Go No-go studies investigating the effect of dopaminergic therapy in PD failed to reveal any significant differences across *on* and *off* states. However, these studies included very small numbers of participants raising suspicion that they were underpowered to detect medication effects. Further, differences in task parameters preclude direct comparisons to our study. Studies using standard versions of the Go No-go task in a larger sample of PD patients is warranted to better investigate these effects.

### Dopaminergic Therapy on Motor vs. Cognitive Impulsivity in PD and Addiction

In paradigms other than the Go No-go task, there is evidence that motor impulsivity is improved by dopaminergic therapy. In a study by Hiebert et al. (2014a), PD patients *off* dopaminergic medications demonstrated *greater* motor impulsivity in the form of enhanced facilitation in the congruent condition of a modified Stroop task that was in fact *normalized* relative to performance of age-matched controls by dopaminergic treatment. This study provided an example of impulsive behavior, specifically motor impulsivity, being *improved*, not worsened, by dopaminergic therapy, supporting the notion that impulsivity is not a unitary construct (Antonelli et al., 2011). Conversely, Stroop interference in the incongruent condition was improved in the *on* relative to *off* states for PD patients in terms of accuracy (Fera et al., 2007) and RT (Caillava-Santos et al., 2015). van Wouwe et al. (2016) found that both levodopa and DAs reduced the magnitude of the Simon effect in PD patients. That is, on medication, PD patients exhibited less interference when they had to respond with a left or right button press that was opposite to the left-right position of a target relative to fixation. These studies in PD patients reveal reduced motor impulsivity related to dopaminergic therapy. These studies are in line with our finding of increased timeout responses in the Go condition for the pramipexole group, perhaps suggesting reduced motor impulsivity in pramipexole-treated healthy controls.

The effect of dopaminergic medications on cognition in PD is complex, with improvements in some functions, and impairments in others (Cools et al., 2001; Rowe et al., 2008; MacDonald and Monchi, 2011; MacDonald et al., 2011). The dopamine overdose hypothesis has been proposed to explain these differential effects (Gotham et al., 1986, 1988; Swainson et al., 2000; Cools et al., 2001; Cools, 2006; Vaillancourt et al., 2013). This view contends that dopamine therapy titrated to the significantly dopamine-depleted DS, overdose the more dopamine-replete brain regions supplied by the relatively-spared VTA (Gotham et al., 1986, 1988; Swainson et al., 2000; Cools et al., 2001; MacDonald and Monchi, 2011; MacDonald et al., 2011; Vaillancourt et al., 2013). Cognitive functions that are mediated by DS such as selective attention (Baunez and Robbins, 1999; MacDonald et al., 2011; de Manzano et al., 2013), response deliberation (Balleine et al., 2007; MacDonald et al., 2011; Hiebert et al., 2014b), overcoming automatic or pre-potent responding (Ali et al., 2009; MacDonald et al., 2011, 2014; Robertson et al., 2015), as well as response inhibition (Zandbelt and Vink, 2010; MacDonald and Monchi, 2011; Wylie et al., 2012) are actually *improved*. In contrast, functions mediated by VTA-innervated brain regions (e.g., VS, hippocampus, prefrontal cortex), particularly reward processing and learning are worsened by dopaminergic therapy, potentially accounting for ICDs (Gotham et al., 1986, 1988; Swainson et al., 2000; Cools et al., 2001, 2007; MacDonald and Monchi, 2011; MacDonald et al., 2013; Vaillancourt et al., 2013; Vo et al., 2014).

In the study by Hiebert et al. (2014a), we interpreted our findings as relating to the fact that DS mediates more considered and less impulsive motor behaviors whereas VTA-innervated brain regions such as VS and medio-frontal regions (e.g., orbitofrontal cortex) underlie reward processing and motivation to pursue appetitive experiences (i.e., cognitive/motivational impulsivity, reflective impulsivity). DS’s role in controlled cognitive and motor responses has been well documented (Hood et al., 2007; Cools et al., 2010; MacDonald et al., 2011; Ness and Beste, 2013; Robertson et al., 2015). Similarly, VS and orbitofrontal cortex are extensively implicated in reward processing and motivation (Balleine et al., 2007; Rowe et al., 2008; Drijgers et al., 2012). Referring to the well-supported dopamine overdose hypothesis (Gotham et al., 1986, 1988; Swainson et al., 2000; Cools et al., 2001; Cools, 2006; Vaillancourt et al., 2013), it is entirely expected that dopaminergic therapy would cause improvements in DS-mediated deliberate responding and thus *reduced* motor impulsivity in PD (Fera et al., 2007; Caillava-Santos et al., 2015; van Wouwe et al., 2016). In contrast, due to overdose of VTA-innervated brain regions that govern these processes, dopaminergic therapy is predicted to impair reward processing and motivation, causing increased motivational/cognitive impulsivity and hence ICDs (Cools et al., 2003; Riba et al., 2008; Voon et al., 2010a; Antonelli et al., 2014).

The finding of decreased motor impulsivity related to dopaminergic therapy is also supported by findings in addiction. Modafinil, a dopamine-enhancing medication, has been shown to increase abstinence in cocaine users (Martínez-Raga et al., 2008; Anderson et al., 2009), to reduce laboratory cocaine self-administration and cocaine dependence in double-blind, placebo-controlled clinical studies (Dackis et al., 2005; Hart et al., 2008). Further, in patients with addiction, modafinil reduces impulsive decision-making (Schmaal et al., 2014) and increases response inhibition (Schmaal et al., 2013). In line with the interpretation that pramipexole might reduce motor impulsivity through activation of DS, using fMRI in substance abusers, modafinil and other medications that have dopaminergic properties, enhance activation of DS (Goudriaan et al., 2013; for a review see Cabrera et al., 2016) and cortical regions to which DS is reciprocally connected such as fronto-parietal cortex (Schmaal et al., 2014), anterior cingulate cortex (Ghahremani et al., 2011; Goudriaan et al., 2013) and supplementary motor cortex (Schmaal et al., 2013). Analogously, using PET in a pre- and post-test design relative to an untreated substance abuser control group, modafinil reduces dopamine ligand binding in bilateral caudate and putamen, reflecting increased endogenous dopamine in these regions related to treatment (Karila et al., 2016). In contrast, modafinil and other dopaminergic medications such naltrexone and bupropion, decrease activation in VTA (Goudriaan et al., 2013) and VS (Myrick et al., 2008; Courtney et al., 2016), which correlates with decreased craving (Myrick et al., 2008; Courtney et al., 2016). These findings are consistent with the PD literature in that dopaminergic therapy decreases activation and reduces functions of VS and other VTA-innervated brain regions and increases activity and improves functions of SN-innervated DS and its cortical partners. Indeed, in a recent review of imaging in substance abuse, Cabrera et al. (2016) summarized that therapies that reduce craving tend to decrease or normalize activation in reward and motivation brain centers (e.g., VS, VTA, amygdala, orbitofrontal cortex), whereas those that correlated with increased sober days and greater self-control, increase activation in cognitive and response control centers (e.g., DS, supplementary motor area, dorsolateral prefrontal cortex).

## Conclusion

In healthy young controls, we found that DA pramipexole had no effect on error rate in the No-go condition. In contrast to its effect on cognitive/motivational impulsivity, pramipexole did not increase motor impulsivity. The finding that pramipexole-treated participants performed significantly more timeout errors in the Go condition relative to placebo controls even hints at the possibility that pramipexole *reduces* motor impulsivity. In line with this possibility, motor impulsivity is reduced by dopaminergic therapy in PD and addiction patients. These effects in PD and addiction are attributed to enhanced DS function related to exogenous dopamine therapy (Cools, 2006; Cabrera et al., 2016).

Effects of dopaminergic therapy on cognition have predominantly been investigated in PD patients. The interpretation of these findings, however, is ambiguous, potentially reflecting main effects of dopaminergic therapy or an interaction between medication and PD pathophysiology. Only by studying effects of dopaminergic therapy in healthy controls can the main effects of these medications be understood. We found that dopaminergic therapy affected Go No-go performance—a measure of motor impulsivity—similarly for healthy controls compared to reported effects in PD patients. This suggests that similar effects of dopaminergic therapy are expected independent of disease stage or severity in PD. Further, these findings have implications for patients treated with dopaminergic therapy for other conditions such as restless leg syndrome (Comella, 2002; Högl et al., 2006; Trenkwalder et al., 2008; Zintzaras et al., 2010; Hornyak et al., 2014) and dystonia (Cloud and Jinnah, 2010; Jankovic, 2013), as well as potential depression (Goto et al., 2006; Papakostas, 2006; Hori and Kunugi, 2012, 2013; Howland, 2012) and addiction (Carroll et al., 1999; Streeter et al., 2005; Ohmura et al., 2011; Makhinson and Gomez-Makhinson, 2014).

## Author Contributions

PAM and KNS designed the experiment; XQY and DG collected data; XQY, DG, AV and PAM performed the analysis; XQY, DG and PAM wrote the manuscript; all authors edited the manuscript and participated in revisions.

## Funding

This research was supported by a Canada Research Chair (CRC) Tier 2 in Cognitive Neuroscience and Neuroimaging and a Natural Sciences and Engineering Research Council of Canada Discovery Grant (Grant #6621) to PAM, a Canada Graduate Scholarship from the Canadian Institute of Health Research (CIHR) to DG, and an Ontario Graduate Scholarship to AV.

## Conflict of Interest Statement

The authors declare that the research was conducted in the absence of any commercial or financial relationships that could be construed as a potential conflict of interest.
